# The *Ustilago maydis* null mutant strains of the RNA-binding protein UmRrm75 accumulate hydrogen peroxide and melanin

**DOI:** 10.1038/s41598-019-47133-4

**Published:** 2019-07-25

**Authors:** Alma Laura Rodríguez-Piña, Margarita Juárez-Montiel, Itzell Eurídice Hernández-Sánchez, Aída Araceli Rodríguez-Hernández, Elihú Bautista, Alicia Becerra-Flora, Edgar Oliver López-Villegas, Juan Francisco Jiménez-Bremont

**Affiliations:** 1 Laboratorio de Biología Molecular de Hongos y Plantas, División de Biología Molecular, Instituto Potosino de Investigación Científica y Tecnológica A.C., San Luis Potosí, Mexico; 2 Laboratorio de Biología Molecular de Bacterias y Levaduras, Escuela Nacional de Ciencias Biológicas, Instituto Politécnico Nacional, México City, Mexico; 3 CONACYT-CIIDZA, Instituto Potosino de Investigación Científica y Tecnológica A.C., San Luis Potosí, Mexico; 4 Central de microscopía, Escuela Nacional de Ciencia Biológicas, Instituto Politécnico Nacional, México City, Mexico; 5 Present Address: CONACyT-Instituto Politécnico Nacional, CEPROBI, Yautepec, Morelos, Mexico

**Keywords:** Fungal genetics, Transcription

## Abstract

*Ustilago maydis* is a dimorphic fungus that has emerged as a model organism for the study of fungal phytopathogenicity and RNA biology. In a previous study, we isolated the *U*. *maydis UmRrm75* gene. The deletion of the *UmRrm75* gene affected morphogenesis and pathogenicity. *UmRrm75* gene encodes a protein containing three RNA recognition motifs. Here we determined that *UmRrm75* has chaperone activity in *Escherichia coli* using the transcription anti-termination assay. Subsequently, we analyzed the growth of Δ*UmRrm75* mutants at 15 °C and 37 °C, observing that mutant strains had reduced growth in comparison to parental strains. *UmRrm75* gene expression was induced under these non-optimal temperatures. Δ*UmRrm75* mutant colonies displayed a dark-brown color at 28 °C, which was confirmed to be melanin based on spectroscopic analysis and spectrometric data. Furthermore, Δ*UmRrm75* mutant strains showed the presence of peroxisomes, and increased H_2_O_2_ levels, even at 28 °C. The Δ*UmRrm75* mutant strains displayed a higher expression of redox-sensor *UmYap1* gene and increased catalase activity than the parental strains. Our data show that deletion of the *UmRrm75* gene results in higher levels of H_2_O_2_, increased melanin content, and abiotic stress sensitivity.

## Introduction

*Ustilago maydis* is a biotrophic fungus that infects maize (*Zea mays*) and teosinte (*Zea perennis*) to produce a disease known as corn smut. *U*. *maydis* is an obligate parasite that can only complete its sexual and infectious cycle in the host plant^1^. This basidiomycete has been widely used for studying the mechanisms of fungal pathogenicity^2,3^. Furthermore, *U*. *maydis* is considered an excellent model for the study of DNA recombination and repair, vesicle trafficking, and RNA biology^3,4^.

RNA binding-proteins (RBPs) are key players in gene expression regulation in all organisms because they mediate rapid changes in expression profile in order to help organisms adapt or overcome environmental changes^5^. RBPs can regulate RNA activity and structure by participating in RNA maturation, nuclear export, stability, transport, and translation^6^. RBPs bind to RNAs with high affinity through RNA-binding domains, modulating the RNA structure. In fungi, the RBPs are involved in growth, development, morphology, pathogenicity, and stress response. In *Aspergillus nidulans*, RBPs play an important role in cell cycle regulation^7^. RBPs in *Saccharomyces cerevisiae* are involved in splicing and mating regulation^5^. In *U*. *maydis*, RBPs participate in filamentation and pathogenicity^8–10^.

In a previous study, we identified the *UmRrm75* gene in *U*. *maydis*, which encodes a protein containing three RNA recognition motifs interspersed by glycine-rich regions^10^. Deletion of the *UmRrm75* gene resulted in several alterations, such as a donut-like morphology, decreased mating and post-mating filamentous growth, and reduced virulence in maize^10^. In this study, we determined that expression of *UmRrm75* gene was induced under thermal stress (15 °C and 37 °C), and showed evidence of RNA chaperone activity. We detected greater sensitivity to temperature stress in Δ*UmRrm75* mutants relative to the parental strains. We noticed that Δ*UmRrm75* mutant strains accumulate a brown-pigment, greater H_2_O_2_ content, and also showed the presence of peroxisomes under both optimal and stress temperatures. Finally, the catalase activity in the Δ*UmRrm75* mutant strains was analyzed, highlighting that the mutants were activating the detoxification system.

## Results

### The UmRrm75 protein exhibits RNA binding activity in *E*. *coli*

**Figure 1:**
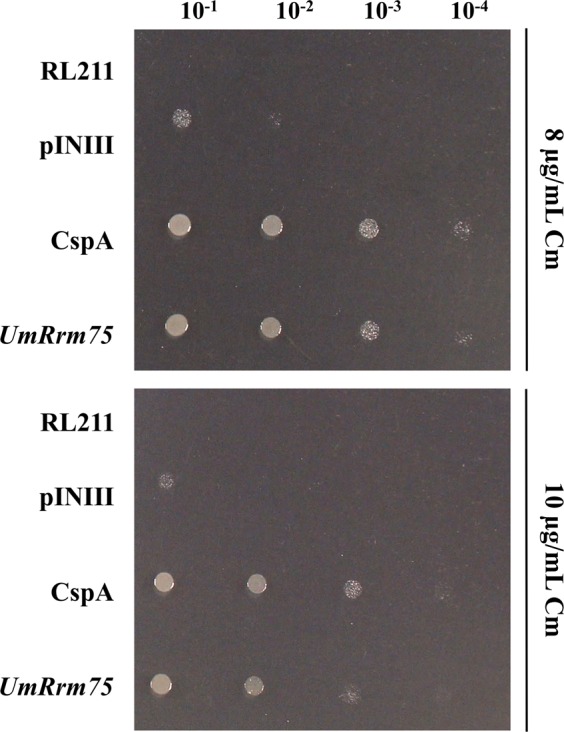
Transcription anti-terminator test of UmRrm75. Anti-terminator assay of UmRrm75 in the RL211 *E*. *coli* strain. Cultures of RL211 (negative control), RL211-pINIII (negative control), RL211-CspA (positive control) and RL211-*UmRrm75* bacterial strains were adjusted to OD_600_ 1.0, and they were spotted in serial dilutions on LB plates with 8 or 10 µg/mL chloramphenicol. Plates were photographed after 48 h of incubation at 37 °C. Data shown are representative of three independent experiments.

In order to analyze the RNA chaperone activity of the UmRrm75 protein, we used the bacterial transcription anti-termination system^11^. The open reading frame (ORF) of the *UmRrm75* gene was cloned into the pINIII expression vector, and transformed into the *E*. *coli* RL211 strain. The RL211 strain contains the chloramphenicol acetyltransferase (cat) gene preceded by a strong loop (ρ-independent trpL) terminator. The melting of this anti-terminator loop confers chloramphenicol (Cm) resistance in *E*. *coli*^11,12^, which makes the system efficient in detecting RNA binding activity. In this assay, we included the RL211 strain expressing *cspA* gene as a positive control of RNA binding activity (RL211-cspA), and the RL211 and RL211-pINIII (empty vector) strains as negative controls. Bacterial growth was evaluated by the drop dilution test in a medium containing 8 and 10 µg/mL Cm. As observed in Fig. 1, the RL211-*UmRrm75* strain achieved growth until the fourth dilution at 8 µg/mL Cm, and up to the third dilution at 10 µg/mL Cm, similar to the observed for RL211-cspA strain. As expected, no growth was observed in the RL211 negative control, and for the RL211-pINIII strain a slight growth was obtained until the first dilution for both Cm concentrations. These results indicate that the UmRrm75 protein was capable of binding and melting RNA secondary structures in *E*. *coli*.

### Deletion of the *UmRrm75* gene affects fungal growth under temperature stress conditions

**Figure 2:**
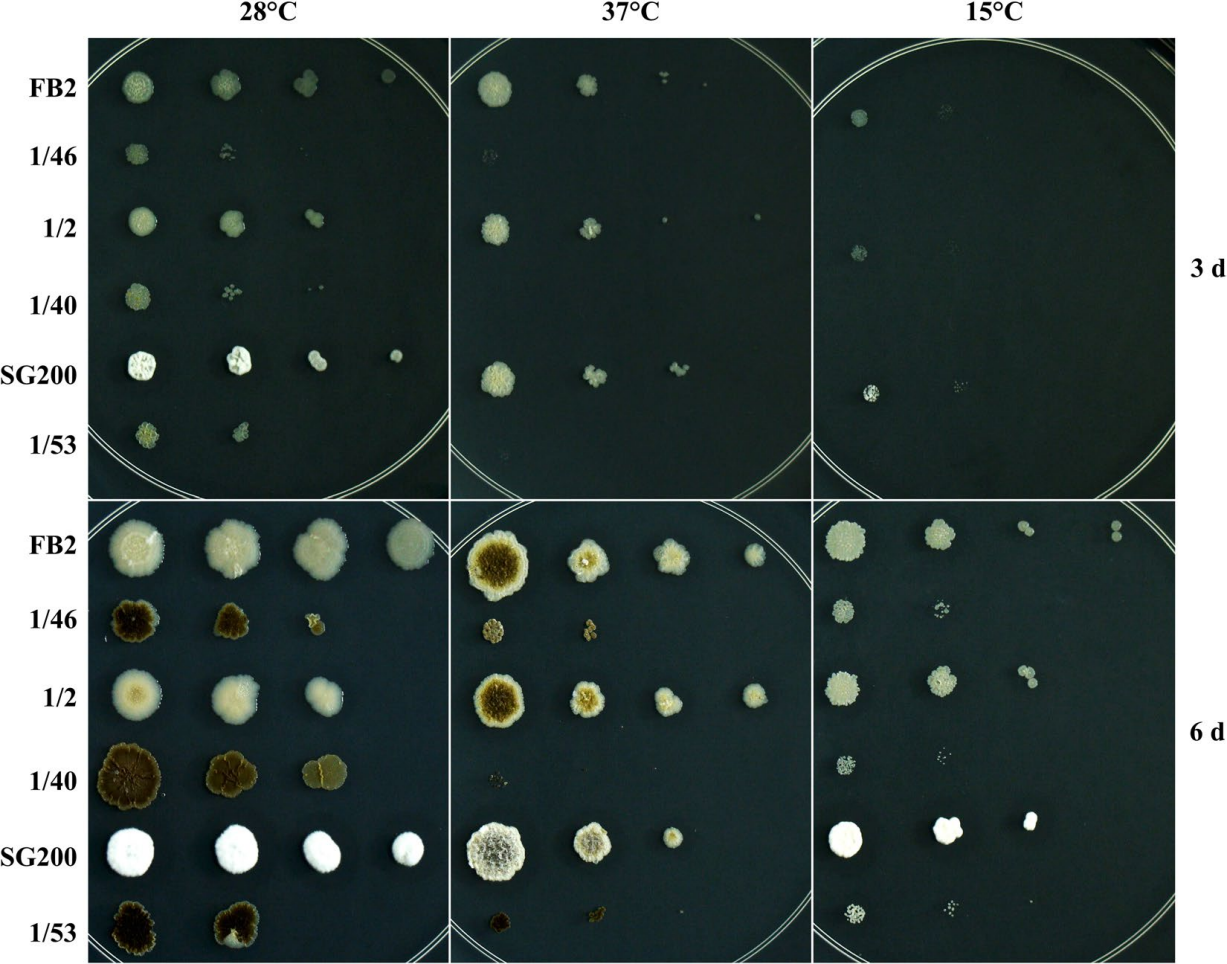
Growth assays of *U*. *maydis* parental and Δ*UmRrm75* mutant strains. Serial dilutions of cultures of *U*. *maydis* were spotted on CM media and incubated at 37°C, 15 °C, and 28 °C (control). Plates were photographed after 3 and 6 days. Data shown are representative of three independent experiments.

We evaluated the effect of non-optimal temperatures on the growth of ∆*UmRrm75* mutant strains (1/46, 1/40 and 1/53), and their respective parental strains (FB2, 1/2, and SG200) (Supplementary Table 1). The *∆UmRrm75* mutants and parental strains were grown on a complete medium (CM) in serial dilutions (1 × 10^2^–1 × 10^5^) for 6 days at 15 °C and 37 °C (non-optimal temperatures), and as a control the temperature 28 °C was used. Although at 28 °C, the *∆UmRrm75* mutants growth was slower than the parental strains, the reduction in growth was more noticeable at 37 °C and 15 °C (Fig. 2). After 3 days of incubation at 37 °C and 15 °C, the Δ*UmRrm75* strains did not show growth, whereas at 28 °C yeast growth was observed until the second dilution (Fig. 2). After 6 days of incubation, null mutants were able to grow until the second dilution at 37 °C or 15 °C, displaying a reduced colony size. These data showed that the Δ*UmRrm75* mutant strains exhibited a slower growth at non-optimal temperatures.

### The *UmRrm75* gene expression is induced under abiotic stress conditions

**Figure 3:**
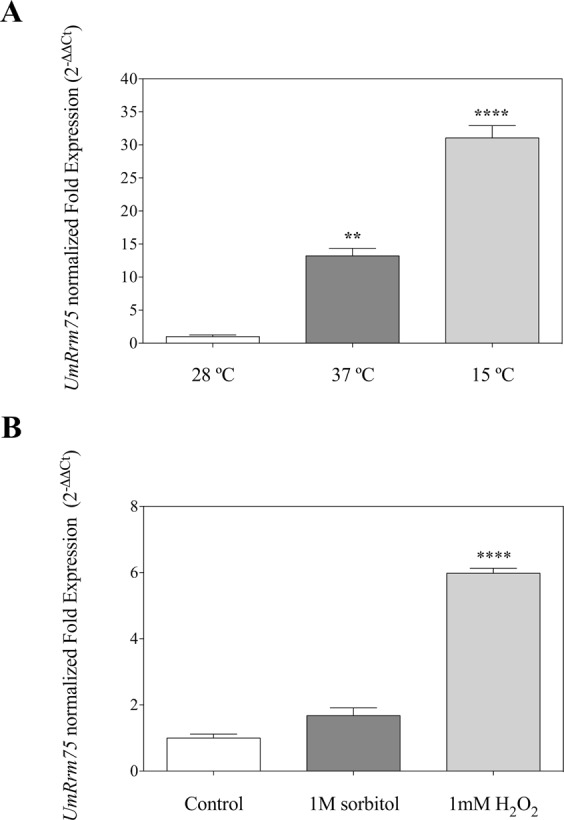
*UmRrm75* gene expression analysis in FB2 parental strain under thermal, osmotic, and oxidative conditions. (**A**) FB2 parental strain grown for 24 h in liquid MM at 15 °C, 28 °C and 37 °C. (**B**) FB2 strain grown for 24 h in liquid MM with 1 M sorbitol or 1 mM H_2_O_2_. Transcript levels of *UmRrm75* were calculated by the qRT-PCR approach. Normalized fold change was calculated comparing the *UmRrm75* gene expression (under stress condition) with control conditions, after normalization to the *UmGAPDH* using the (2^−ΔΔCt^) method. Analyses were performed in triplicate. Data represent the mean ± SEM. One-Way Analysis of Variance (ANOVA) and Tukey’s post-test analyses were performed. Significant differences are marked with an asterisk (P < 0.05).

We evaluated the expression of the *UmRrm75* gene under several abiotic stresses in parental strain FB2. The FB2 strain was grown in liquid minimal medium (MM) at 15 °C, 28 °C, and 37 °C for 24 h. Expression levels were determined by qRT-PCR and normalized against the optimal condition (28 °C). *UmRrm75* gene was expressed at very high levels under non-optimal temperatures, 13.2-fold at 37 °C, and 31-fold at 15 °C, in contrast to those levels observed in the control condition at 28 °C (Fig. 3A). We also analyzed the *UmRrm75* expression levels in FB2 strain grown in liquid MM supplemented with 1 M sorbitol or 1 mM H_2_O_2_ for 24 h. We observed an increase in *UmRrm75* expression levels of 1.7-fold with sorbitol and 6-fold with H_2_O_2_ treatments (Fig. 3B). These results revealed that *UmRrm75* gene was mainly regulated by thermal stress conditions.

### The ∆*UmRrm75* mutant strains accumulate a dark brown pigment under optimal and non-optimal temperatures

We observed that *UmRrm75* null mutant colonies grown under optimal conditions (28 °C) exhibited a dark brown pigmentation after 6 days of growth (Fig. 2). This pigment was also observed when the Δ*UmRrm75* strains were grown at 37 °C. In the parental strains, the brown pigmentation was only observed under 37 °C stress treatment (Fig. 2). These data show that the Δ*UmRrm75* mutant colonies exhibit an accumulation of a dark-brown pigment, even under optimal temperature conditions.

### The *∆UmRrm75* mutant strains accumulate melanin

**Figure 4:**
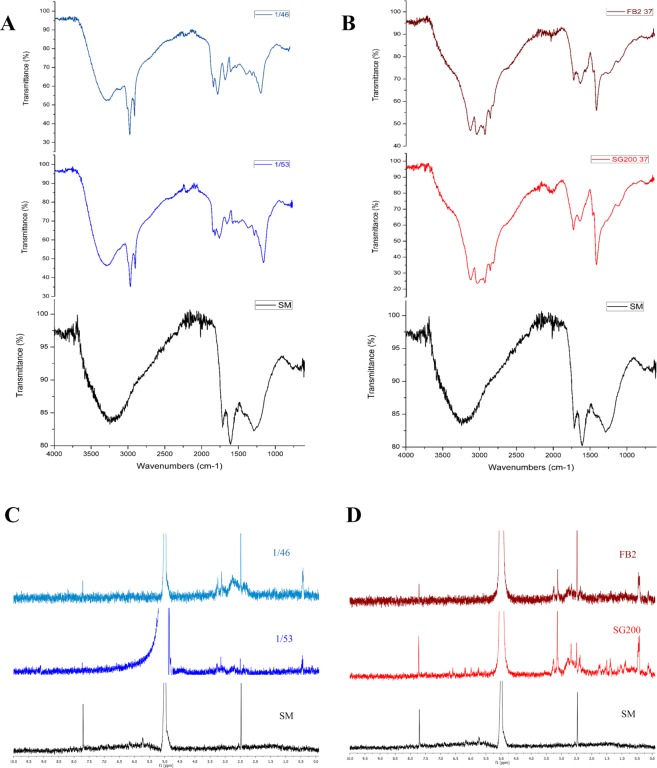
IR and ^1^H NMR spectra analysis of pigments from Δ*UmRrm75* null mutant and parental strains. (**A**) Infrared spectra of melanin extracted from 1/46 and 1/53 null mutant strains at 28 °C (**B**) Infrared spectra of melanin extracted from FB2 and SG200 parental strains at 37 °C. (**C**) ^1^H NMR spectra of melanin extracted from 1/46 and 1/53 null mutant strains at 28 °C. (**D**) ^1^H NMR spectra of melanin extracted from FB2 and SG200 parental strains at 37 °C. All spectra were compared with synthetic melanin as a reference.

In fungi, the accumulation of melanin and other non-enzymatic metabolites are part of the mechanisms of protection against oxidizing agents^13^. We performed several chemical tests to determine if the dark-brown pigment accumulated in ∆*UmRrm75* strains (1/46 and 1/53), and their respective parental strains (FB2 and SG200) corresponded to melanin compounds. Our first physicochemical data revealed that the pigment accumulated in ∆*UmRrm75* strains displayed typical characteristics of melanin such as brown coloration, insolubility in organic compounds, and was soluble at 100 °C in KOH alkaline solution (Supplementary Table 2). In the second approach spectroscopic methods were employed, which agreed with the melanin nature of *U*. *maydis* pigments; for example, UV-Vis spectroscopy revealed maximum absorption between 210–220 nm for ∆*UmRrm75* mutants at 28 °C and parental strains at 37 °C (Supplementary Fig. 1). Infrared analyses showed that ∆*UmRrm75* mutants at 28 °C, and parental strains at 37 °C, have bands representing phenolic groups (3400-3100 and 1260-1240 cm^−1^), methyl or methylene groups (2980-2850 cm^−1^), and -NH groups (3300-3260 and 1650-1630 cm^−1^). Unique bands between 2980-2850 cm^−1^ were observed in the pigment purified from *UmRrm75* mutants. These peaks could be explained as being specific to the *U*. *maydis* melanin (Fig. 4A–B). Finally, the analysis through ^1^H NMR of melanins from *∆UmRrm75* at 28 °C and parental strains at 37 °C displayed two signals at δ_H_ 7.70 and 2.48 ppm, which can be assigned to CH=C and –NH groups of the indole moiety. Additional signals at δ_H_ 3.24, 3.13, and 0.45 ppm were detected in *∆UmRrm75* mutant’s melanin (Fig. 4C,D). The melanin content in parental strains at 28 °C and 37 °C was not detected by subsequent spectrometric analyses. The ESI-MS analysis revealed that melanins produced by *UmRrm75* mutants at 28 °C were closely similar to those obtained for synthetic melanin with fragment losses of 150 amu (Supplementary Fig. 2). These spectroscopic and spectrometric data indicate that the pigment produced by Δ*UmRrm75* mutant and parental strains under stress conditions was melanin of the eumelanine type, consisting mainly of the 5,6-dihydoxyindole (DHI) building block^14,15^.

### The Δ*UmRrm75* mutant strains accumulate H_2_O_2_

**Figure 5:**
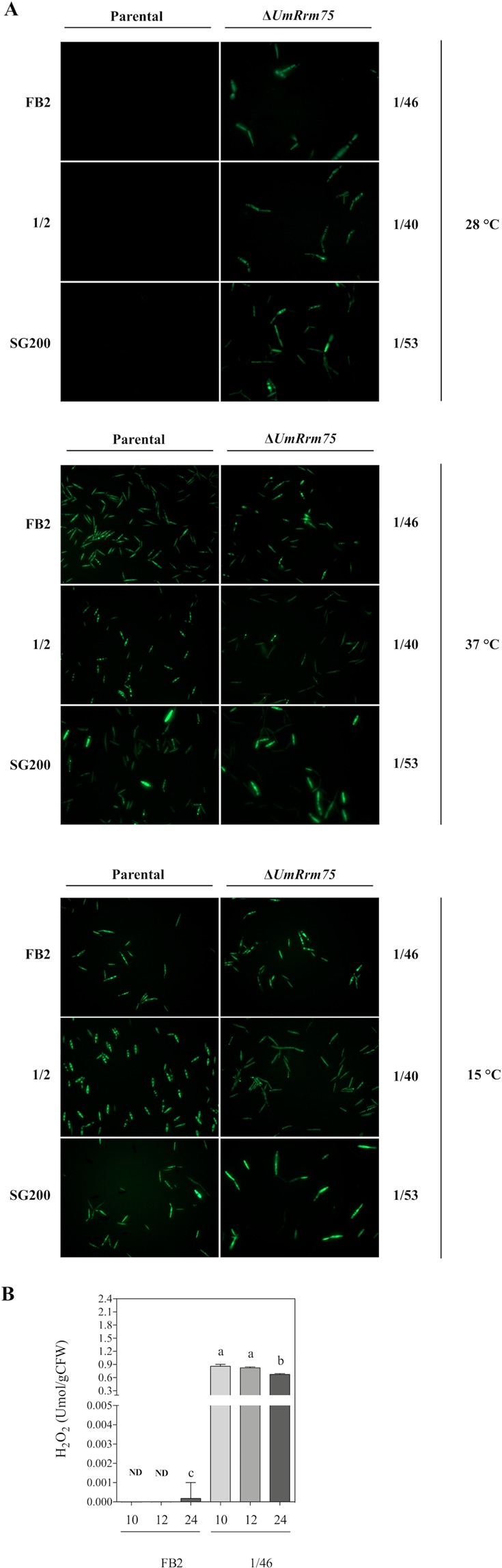
Hydrogen peroxide detection in ∆*UmRrm75* mutant and parental strains. (**A**) FB2, 1/2 and SG200 parental strains, and their respective mutant strains (1/46, 1/40 and 1/53) were grown in liquid MM with DCFH2-DA for 4 h at 15 °C, 28 °C and 37 °C. Images were taken with a 40x oil-objective. Data shown are representative of results of three biological replicates. (**B**) H_2_O_2_ quantification was performed using KI in *U*. *maydis* cells. FB2 parental and 1/46 mutant strains were grown in liquid MM for 10, 12, and 24 h. Data are reported as μmol/gCFW. Data are means ± SEM from three biological replicates (*n* = 3). Different letters indicate a significant difference according to One-Way Analysis of Variance (ANOVA) and Tukey’s post-test analysis.

**Figure 6:**
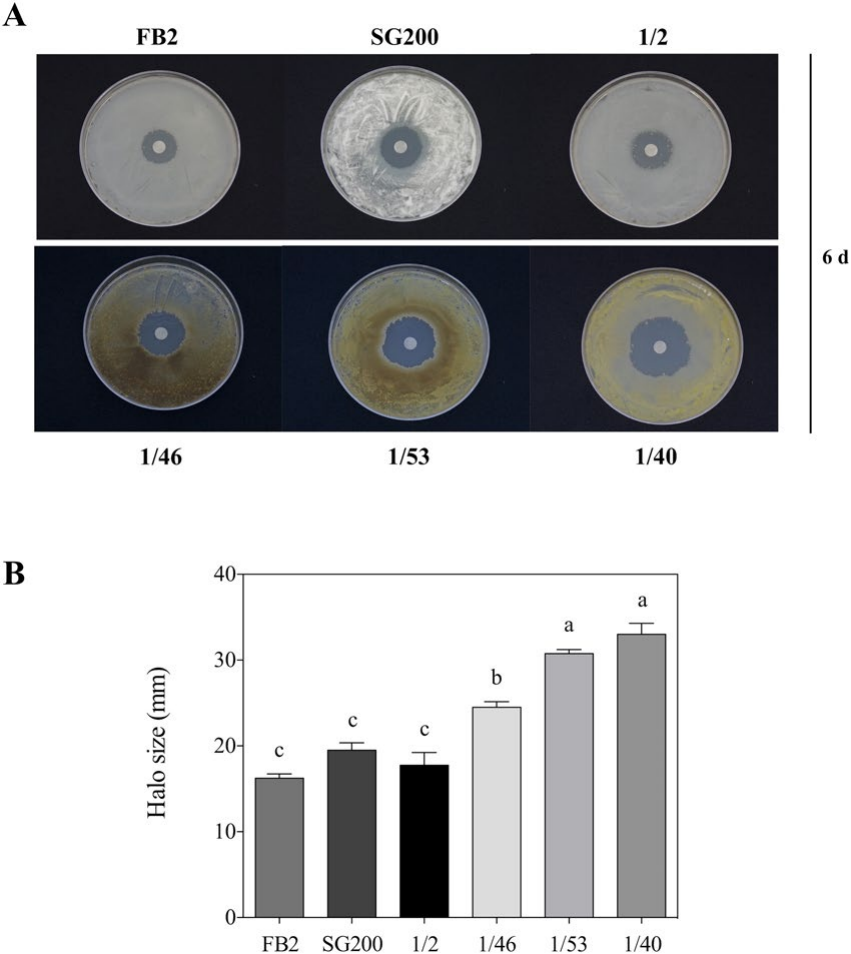
Sensitivity of ∆*UmRrm75* mutant and parental strains to H_2_O_2_. (**A**) Sensitivity assays was assessed in an agar diffusion test with a filter soaked with 1uL H_2_O_2_ (30% v/v) was placed on agar plate. (**B**) Halo size was quantified as the diameter of each ∆*UmRrm75* mutant and parental strains grown for 6 days. Data represent the mean ± SEM (*n* = 4) and differences at P < 0.05 were considered significant. The different letters indicate a significant difference according to One-Way Analysis of Variance (ANOVA) and Tukey’s post-test analysis.

The presence of melanin in Δ*UmRrm75* mutants incubated at 28 °C or 37 °C, and in parental strains at 37 °C, suggested changes in H_2_O_2_ content. We evaluated H_2_O_2_ production using 2′,7′-dichlorofluorescein diacetate dye using Epi-fluorescence microscopy. Under optimal growth conditions (28 °C), H_2_O_2_ signal was clearly observed as a green fluorescent signal in Δ*UmRrm75* mutant strains, while in parental strains no signal was detected (Fig. 5A). However, when cells were grown at 15 °C or 37 °C, the green fluorescent signal was observed in both parental and Δ*UmRrm75* strains (Fig. 5A). H_2_O_2_ was quantified in FB2 parental and 1/46 mutant strains grown for 10, 12 and 24 h at 28 °C, respectively. Our data indicated high levels of H_2_O_2_ in the 1/46 mutant for all tested times, whereas the FB2 showed basal or no detectable H_2_O_2_ (Fig. 5B). In addition, Δ*UmRrm75* mutants were subjected to exogenous H_2_O_2_ treatment in an agar diffusion test for 6 days at 28 °C. We observed that the growth inhibition halo was wider in all Δ*UmRrm75* strains in comparison to the parental strains (Fig. 6A). After 6 days of the H_2_O_2_ diffusion test, the characteristic brown pigment was only observed in the Δ*UmRrm75* strains (Fig. 6B). Our results indicated that Δ*UmRrm75* mutant strains accumulate H_2_O_2_, even under optimal conditions, which made them more sensitive to the application of exogenous H_2_O_2_.

### The Δ***UmRrm75*** mutants show accumulation of peroxisomes

**Figure 7:**
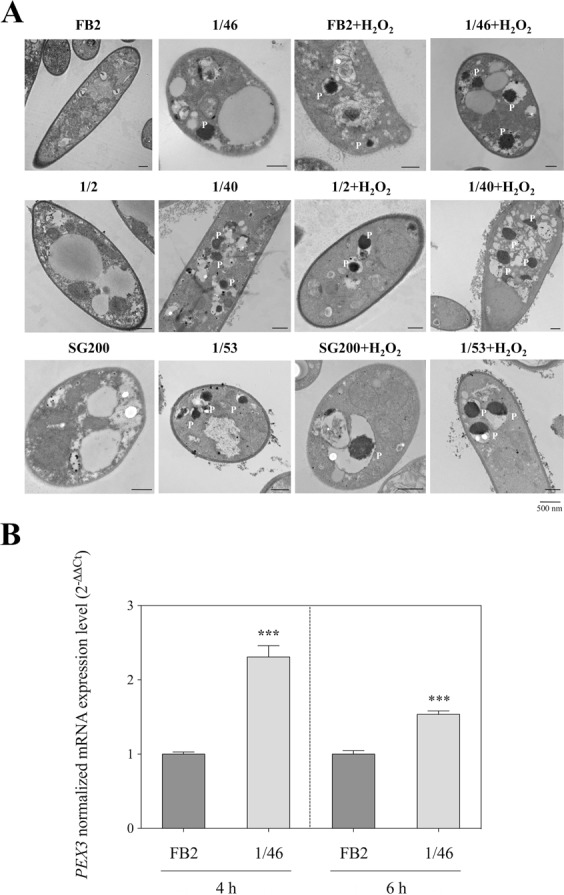
Detection of peroxisomes by the DAB method, and quantification of *UmPex3* gene expression in Δ*UmRrm75* mutant and parental strains. (**A**) TEM images of FB2, 1/2 and SG200 parental strains and 1/46, 1/40 and 1/53 mutant strains grown in liquid MM supplemented with 0 or 1 mM H_2_O_2_. Peroxisomes containing DAB reaction product are marked by the letter P. Bar correspond to 500 nm. (**B**) Transcript expression analysis of *UmPex3* gene in FB2 parental and 1/46 mutant strains. Normalized fold change was calculated comparing the *UmPex3* gene expression in 1/46 mutant with FB2 parental, after normalization to the *UmGAPDH* using the (2^−ΔΔCt^) method. Analyses were performed in triplicate. An unpaired t-test was performed. Data represent the mean ± SEM. Significant differences are marked with an asterisk (P < 0.0001).

Peroxisomes play an important role in the protection of cells from reactive oxygen species^16^. We focused on peroxisomes visualization of Δ*UmRrm75* mutant and parental strains using an ultrastructural cytochemical staining approach (DAB-oxidation). The *∆UmRrm75* mutants and parental cells were grown on minimal medium (MM) at 28 °C for 24 h. Images of cells from parental strains did not display the staining signal from the DAB-oxidation reaction (Fig. 7A). Conversely, cells from the Δ*UmRrm75* mutant strains clearly showed a positive DAB-oxidation reaction in peroxisomes (Fig. 7A). As a control, we also analyzed the effect of the exogenous application of 1 mM H_2_O_2_ on DAB-staining in parental and mutant strains. As expected, both mutant and parental cells presented an intense signal of the DAB-reaction product in the peroxisomes (Fig. 7A). Subsequently, we quantified the transcript expression of the peroxisome membrane biogenesis factor *UmPex3* gene^17^ in FB2 parental and 1/46 mutant strains. Both strains were grown in liquid MM for 4 and 6 h at 28 °C. Our results revealed that the *UmPex3* gene was induced in the 1/46 mutant 1.3-fold at 4 h and 0.5-fold at 6 h relative to FB2 (Fig. 7B). These results suggest a peroxisome proliferation in Δ*UmRrm75* mutant strains that can be explained as a consequence of H_2_O_2_ accumulation.

### Δ*UmRrm75* mutant strains show increased catalase activity

**Figure 8:**
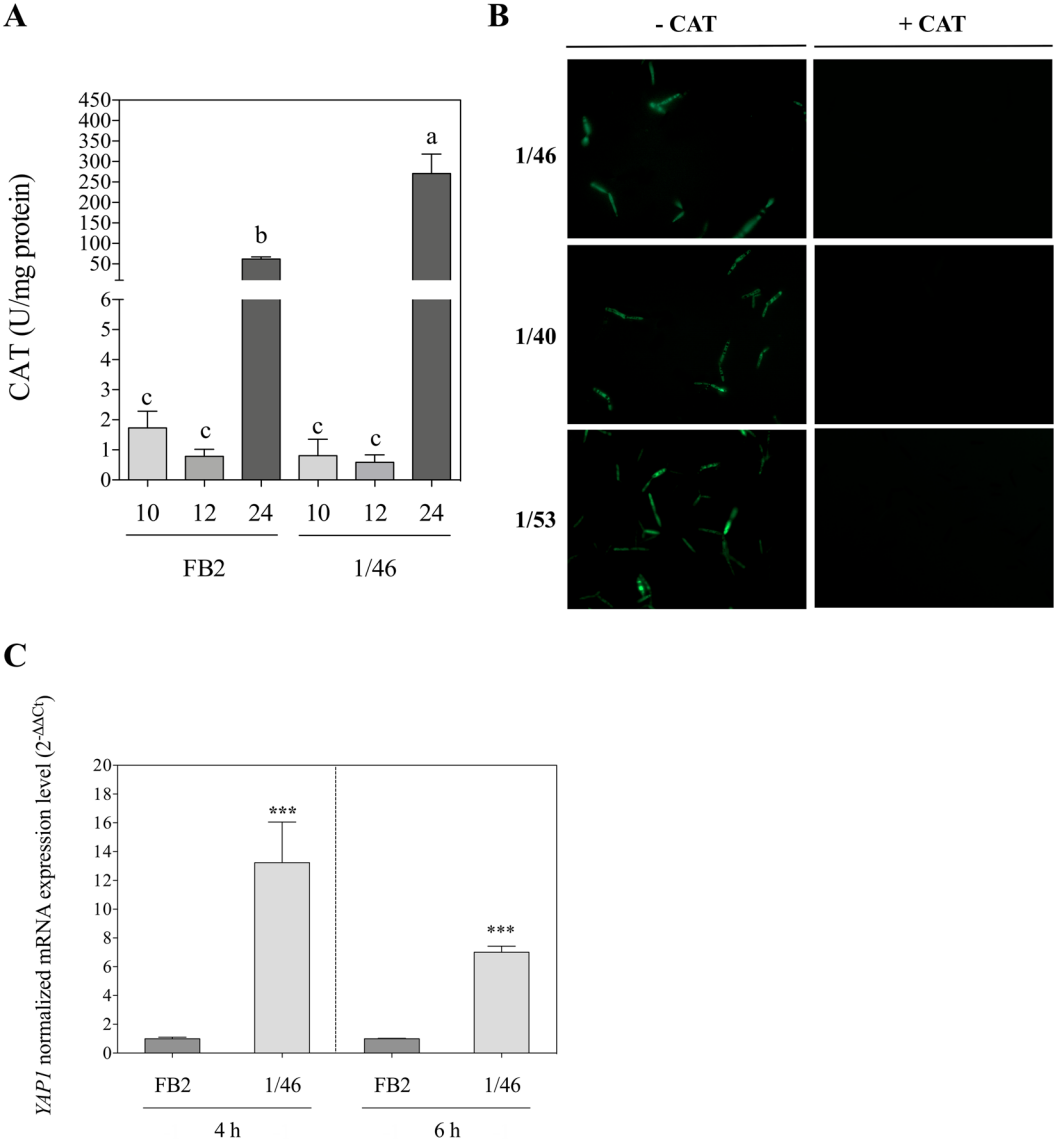
Catalase treatment and enzyme activity, and *UmYap1* expression in the Δ*UmRrm75* mutant and parental strains. (**A**) Determination of catalase (CAT) activity in FB2 parental and 1/46 mutant strains at 10, 12 and 24 h. Data are means ± SEM from three biological replicates (n = 3). The different letters indicate a significant difference according to One-Way Analysis of Variance (ANOVA) and Tukey’s post-test analysis (P < 0.05). (**B**) Exogenous application of CAT in Δ*UmRrm75* mutant strains. Cultures grown in liquid MM with DCFH2-DA at 28 °C; Left panel (without CAT), and right panel (250 U/mL CAT). Images were acquired on a fluorescence microscope at 40× (oil-objective) magnification. One representative microscopy image of DCFH2-DA fluorescence is shown from three biological replicates. (**C**) Transcript level analysis of *UmYap1* gene in FB2 parental and 1/46 mutant strains. Normalized fold change was calculated by comparing the *UmYap1* gene expression in 1/46 mutant with the FB2 parental, after normalization to *UmGAPDH* using the (2^−ΔΔCt^) method. Analyses were performed in triplicate. Unpaired t tests were performed and data represent the mean ± SEM. Significant differences are marked with an asterisk (*P* < 0.0001).

According to the previous data, we quantified the catalase (CAT) activity in 1/46 mutant and FB2 parental strains at 28 °C grown for 10, 12, and 24 h. No changes in CAT activity were observed between the 1/46 mutant and FB2 at 10 or 12 h. After 24 h of growth, CAT activity was increased in the 1/46 mutant (3-fold) in comparison to the FB2 strain (Fig. 8A), showing that the unusual H_2_O_2_ accumulation in the 1/46 mutant strain is activating the detoxification system when grown at an optimal temperature. These data suggest that the sustained oxidative stress in 1/46 mutant is due to the increased levels of H_2_O_2_ generation.

### Exogenous application of catalase alleviates H_2_O_2_ accumulation in Δ*UmRrm75* mutant strains

As a defense mechanism during oxidative stress, cells produce antioxidant enzymes such as superoxide dismutase (SOD) and catalase (CAT). These enzymes are responsible for converting reactive oxygen species (ROS) into harmless products^18,19^. In order to explore if the application of exogenous CAT enzyme may reduce H_2_O_2_ accumulation in Δ*UmRrm75* mutants under control conditions (28 °C), we incubated null mutant cells with 0 or 250 U/mL of CAT with 2′,7′-dichlorofluorescein diacetate dye. We did not observe fluorescent signals due to H_2_O_2_ accumulation from Δ*UmRrm75* cells after the application of 250 U/mL CAT (Fig. 8B). Thus, this result confirmed H_2_O_2_ accumulation in Δ*UmRrm75* mutants, and revealed that this accumulation can be scavenged by the application of exogenous CAT.

### *UmYap1* gene is induced in Δ*UmRrm75* mutant (1/46) under optimal conditions

The *UmYap1* transcription factor plays an essential role in the detoxification of *U*. *maydis* cells by acting as a redox sensor^20^. In order to evaluate the expression of the *UmYap1* gene in response to H_2_O_2_ accumulation, we analyzed the FB2 parental and 1/46 mutant strains using qRT-PCR. Both strains were grown in liquid MM at 28 °C for 4 and 6 h. The results showed that the *UmYap1* gene was highly induced in the 1/46 mutant: 13-fold at 4 h and 7-fold at 6 h in comparison to FB2 strain (Fig. 8C). The high transcriptional levels of *UmYap1* supported the notion that the redox sensor was not affected in the Δ*UmRrm75* mutant, and that the mutant is working to counteract the accumulation of H_2_O_2_ under normal growth conditions.

## Discussion

Our data showed that the UmRrm75 protein had RNA chaperone activity in an *E*. *coli* heterologous system, providing relevant evidence about the possible role of this protein in *U*. *maydis* as a RNA chaperone. In bacteria, there is evidence that cold shock proteins (CSPs) with RNA binding domains have RNA chaperone activity under stress conditions. It has been reported that CSPs play important roles in response to low-temperature, post-transcriptional machinery regulation, adaptation, and survival^21,22^. In plants, there are homologs of CSPs, which contain an N-terminal cold shock domain and also glycine rich domains^23^. These glycine-rich RNA-binding proteins are related to freezing stress tolerance in Arabidopsis^24^. In fungi, the RNA binding proteins (RBPs) have been related to growth, development, morphology, pathogenicity processes, and stress response^25–27^. It is well known that many fungi are able to adapt and overcome extreme temperatures^28,29^. However, the molecular mechanisms, and particularly the role of RNA binding proteins under temperature stress, have not been fully explored^27,30^. Here, we analyzed the growth capacity of the Δ*UmRrm75* mutant strains under stress temperatures, 15 °C and 37 °C. We found that Δ*UmRrm75* mutant strains were affected in their growth capacity (even at an optimal temperature of 28 °C) in contrast to the parental strains. These data correlated with the induction of *UmRrm75* gene in the FB2 parental strain that was subjected to 15 °C and 37 °C. Fang & St Leger^27^ reported that two RNA binding proteins (Crp1 and Crp2) of *Metarhizium anisopliae* fungus were also capable of melting RNA secondary structure in an *E*. *coli* heterologous system. Moreover, when *M*. *anisopliae* was subjected to abiotic stress conditions and non-optimal temperatures stress, a high expression level of *Crp1* was observed under all stress conditions.

The accumulation of dark-brown pigments in many fungi is associated to environmental stress response^31–34^. Δ*UmRrm75* mutant strains after 6 days at 28 °C accumulated a dark-brown pigment. When strains were challenged to heat stress (37 °C), parental strains also showed accumulation of this pigment. At a low temperature (15 °C), no pigmentation was observed, neither in parental nor in Δ*UmRrm75* mutant strains. Therefore, this pigmentation correlated with the deletion of the *UmRrm75* gene, and also as a response to heat.

Dark-brown pigments produced by Δ*UmRrm75* mutants and parental strains were characterized by spectroscopic and spectrometric analyses. Our data showed that this pigment has the same chemical properties of those reported for fungal melanins^35^. In addition, UV-Vis spectra of Δ*UmRrm75* mutant at 28 °C and parental strains at 37 °C displayed absorption profiles similar to a synthetic melanin employed as a reference. The log of optical density of melanin solution when plotted against wavelength produces a linear curve with negative slopes^35,36^. The IR spectrum of synthetic melanin showed the same bands produced by Δ*UmRrm75* mutant at 28 °C and parental strains at 37 °C (except the bands between 2980-2850 cm^−1^), and also to those observed in other melanins isolated from fungi and plants^15,37^. The ESI-MS analyses of melanin from Δ*UmRrm75* mutant strains were carried out in an *m/z* ranging from 100–2000 amu. Mass spectra showed molecular ions at *m/z* 1679 (melanin reference at *m/z* 1677), and subsequent fragments with losses of multiples of 150 amu (*m/z* 1529, 1379 and 1231), suggesting that 5,6-dihydroxyindole (DHI) is the main building block for this melanin. This fragmentation pattern was consistent with those described for other melanins, i.e. those containing 3,4-dihydoxyphenylalanine and *p*-coumaric acid as monomeric units^14,15^. These data were supported by the ^1^H NMR spectra of melanins from *∆UmRrm75* mutants and parental strains. The signals at *δ*_H_ 7.70 and 2.48 ppm also indicated that melanins consisted of 5,6-dihydroxyindole (DHI) as the main building block. Additional signals at *δ*_H_ 3.24, 3.13, and 0.45 were observed. The first two signals could be attributed to other –NH groups of building blocks described for melanins, such as pyrrole-2,3-dicarboxylic acid or pyrrole-2,3,5-tricarboxylic acid^38^, and the last signal could be attributed to methylene groups of another kind of pyrroles moiety^39^. In summary, spectral comparison of melanins derived from *∆UmRrm75* mutant and parental strains with those described for natural and semi-synthetic melanins from fungal origin, isolated from *Lachnum* species, showed a great similarity and supported our data on the structure of melanins from *U*. *maydis*^37,40^.

Melanin is described as a dark-brown pigment formed by polymeric macromolecules of hydrophobic character with a negative charge, such as phenolic or indole rings^41–43^. Melanin is a multifunctional pigment that is found in all biological kingdoms, and is involved in the defense against environmental stresses such as ultraviolet (UV) light, oxidizing agents and ionizing radiation^44^. In fungi, it is well documented that melanin contributes to the ability of survival in harsh environments, and tolerance to desiccation and extreme temperatures, as well as chemo-protector absorbing free radicals, protecting against oxidative stress and UV radiation^45–48^. Particularly, Rita & Pombeiro-Sponchiado^49^ reported that the melanin from *Aspergillus nidulans* has a potential activity as HOCl and H_2_O_2_ scavenger. Our data show that the *UmRrm75* gene deletion affects H_2_O_2_ and melanin content, which suggest that Δ*UmRrm75* mutants are stressed even under normal conditions.

In fungi, like in many other aerobic organisms, one of the first cell detoxification responses is against ROS accumulation, which includes an increase in the activities of the principal antioxidant enzymes, such as superoxide dismutase (SOD) and catalase (CAT), which play key roles in ROS scavenging^50–52^. Herein, we reported that CAT activity was higher in the 1/46 mutant strain than FB2 parental strain. The increase in CAT activity in the 1/46 mutant strain could indicate that the null mutant cells are trying to degrade the H_2_O_2_ over-accumulation. Despite this induction in CAT activity, the 1/46 mutant was not able to maintain the H_2_O_2_ homeostasis inside cells. Sokolovsky & Belozerskaya^53^ suggested that higher CAT and SOD activities in fungus are associated with resistance to oxidative stress factors such as H_2_O_2_, which is the most stable species of ROS found inside the cell. However, when we applied exogenous CAT enzyme to the Δ*UmRrm75* mutant strains, H_2_O_2_ levels were reduced.

It is interesting that we observed peroxisomes in mutant strains when they were grown at 28 °C; this phenotype was only achieved in the parental strains when exogenous H_2_O_2_ was added. Furthermore, we analyzed the *UmPex3* gene, which encodes a peroxisomal membrane biogenesis factor^17^. We found that *UmPex3* gene showed higher transcript levels in the 1/46 mutant than FB2, which suggests *de novo* peroxisome biogenesis is occurring in the Δ*UmRrm75* mutant strains. The DAB staining evidences that H_2_O_2_ is accumulating inside the peroxisomes. Schrader and Dariush^54^ describe that this organelle participates in both the production and the scavenging of ROS, particular H_2_O_2_. Peroxisomes can proliferate in response to nutritional and extracellular environmental stimuli; this response is usually accomplished by the induction of peroxisomal enzymes^55^, as was observed in our study. We propose that this peroxisomal proliferation in Δ*UmRrm75* mutant strain is a part of the *U*. *maydis* scavenging response to ROS accumulation.

In *U*. *maydis*, the transcription factor *UmYap1* controls the cells detoxification pathway; this gene functions as a redox sensor, and is essential for virulence^20^. Finally, we studied how the *UmYap1* gene is expressed in the Δ*UmRrm75* (1/46) mutant strain. Thus, the high expression level of *UmYap1* in the 1/46 mutant confirms that the detoxification system is active, and could be regulating H_2_O_2_ accumulation, but is not enough to alleviate the oxidative stress exhibited in the Δ*UmRrm75* mutant strains.

In summary, this study provides novel data about the UmRrm75 protein. The transcription anti-termination assay demonstrated that UmRrm75 has an RNA chaperone activity. We found that Δ*UmRRm75* mutant strains accumulate H_2_O_2_, peroxisomes, and melanin. Consequently, Δ*UmRrm75* mutant strains showed an increased level of *UmYap1* transcript and CAT activity. These findings could explain the previously observed phenotype of slow growth and reduced virulence in the *Ustilago maydis* Δ*UmRRm75* mutant strains^10^.

## Materials and Methods

### Strains, media and growth conditions

The *Ustilago maydis* FB2, 1/2 and SG200 parental and *∆UmRrm75* 1/46, 1/40 and 1/53 null mutant strains^10^, and *Escherichia coli* RL211 strain were used (genotypes are listed in Supplementary Table 1). For *U*. *maydis* growth, the complete medium (CM; 0.1% yeast extract, 0.5% casein peptone, 6.25% salt solution, 0.15% KNO_3_, 1.5% agar and 1% glucose), minimal medium (MM; 2% glucose, 3% KNO_3_ and 6.25% salt solution)^56^, and YEPD medium (1% yeast extract, 2% peptone, and 2% glucose) were used. For *E*. *coli* RL211 growth, Luria broth (LB; 1% peptone, 0.5% yeast extract, 1% NaCl, 500 μM IPTG) was used. These strains were stored at −70 °C in 50% glycerol (v/v). The *U*. *maydis* strains were recovered in YEPD at 28 °C, and the *E*. *coli* strain was recovered in LB medium at 37 °C.

### Thermal stress assay

The *U*. *maydis* parental and *UmRRm75* null mutant strains were grown overnight in YEPD medium. Cells were adjusted to an OD_600_ of 0.3 with fresh YEPD medium, and incubated at 28 °C until an OD_600_ of 0.8–1.0 was reached. Cells were collected by centrifugation, and pellets were washed twice with sterile distilled water. Subsequently, 2 µL of each suspension were spotted at four serial dilutions (1 × 10^2^–1 × 10^5^) in Petri dishes containing solid CM. The inoculated plates were incubated at 15 °C, 28 °C or 37 °C for 3 or 6 days. Images shown are representative of the experiment conducted with three biological replicates. This experiment was repeated at least 3 times with similar results.

### *In vivo* transcription anti-terminator assay

The open reading frame (ORF) of *UmRrm75* gene was amplified by PCR using Phusion High-fidelity DNA polymerase (ThermoFisher, Carlsbad, CA, USA) and cloned between the *Xba*I/*Bam*H1 restrictions sites present in the pINIII plasmid. The pINIII:*UmRrm75* construct was confirmed by sequencing, and transformed in the *E*. *coli* RL211 mutant strain^11^. As a positive control, RL211 strain was transformed with the pINIII-*CspA* plasmid, and as a negative control the pINIII empty vector was used. The RL211 strains carrying the various vectors were grown in LB liquid medium, and then spotted in serial dilutions (1 × 10^2^–1 × 10^5^) on LB plates supplemented with 8 µg/L or 10 µg/L chloramphenicol (Cm). Plates were incubated at 37 °C for 72 h. Photographs shown are representative of the experiment conducted with three biological replicates. This experiment was repeated at least 3 times with similar results.

### Expression analysis of *UmRrm75* transcript under abiotic stress conditions

For all treatments, the FB2 parental strain was grown overnight in YEPD liquid medium at 28 °C. Then, the cell culture was adjusted to an OD_600_ of 0.3 using fresh liquid MM. For the thermal stress assay, cells were grown at 15 °C, 28 °C or 37 °C for 24 h. For osmotic and oxidative stresses, 1 M sorbitol and 1 mM H_2_O_2_, were independently added to the FB2 parental cells culture and then subsequently grown for 24 h. After cells were collected, pellets of each condition were stored at −70 °C for subsequent RNA extraction.

### Analysis of *UmYap1* and *UmPex3* transcripts in parental and null mutant strains

The FB2 parental and 1/46 null mutant strains were grown at 28 °C in liquid MM and collected at 4 and 6 h. Then, the pellets for each strain were stored at −70 °C for a subsequent RNA extraction. The RNA extraction method was conducted as described by Collart and Oliviero^57^. The genomic DNA was removed using TURBO DNAse enzyme (Ambion, Austin, TX, USA) according to the manufacturer’s protocol. For synthesis and quantification of cDNA, the One-Step Kit and Power SYBR Green RNA-to-CT kit (Applied Biosystems, USA) were used. The qRT-PCR was performed as described previously in Rodríguez-Hernández *et al*.^58^ and Ortega-Amaro *et al*.^59^. The *UmRrm75*, *UmPex3*, and *UmYap1* gene expression were analyzed by the 2^−ΔΔCT^ method^60^ and the data were normalized against the *UmGAPDH* gene. The designed primers are listed in Supplementary Table 3. For each sample, three biological replicates (*n* = 3) were analyzed with their respective technical replicate.

### Extraction and isolation of melanin

Parental (FB2 and SG200) and Δ*UmRrm75* mutant (1/46 and 1/53) strains of *U*. *maydis* were grown in liquid MM at 37 °C and 28 °C respectively, for 10 days. Afterwards, each culture was centrifuged and washed twice with sterile deionized water. Each cellular pellet was dissolved in 1 M NaOH and heated to 120 °C for 20 min; then acidified with 6 M HCl and heated to 100 °C for 3 h, and then centrifuged for 10 min. The pellet was dissolved in 0.1 M KOH. Concentrated HCl was added to the aqueous portion to precipitate the brown pigment. The precipitate material was washed with distilled water and dried in a SpeedVac Concentrator (SAVANT, SPD131DDA) with a refrigerated vapor trap (RVT405DDA) at RT for 2 h. The obtained powder was used for spectroscopic and spectrometric analysis.

### Experimental procedures for spectroscopic and spectrometric characterization of melanins

UV-Vis spectra of aqueous solution of brown pigment at a concentration of 10 μg/mL in 0.1 M KOH were recorded using a Thermo Scientific Aquamate 9423AQA2700E UV-Vis Spectrophotometer in the wavelength range 200–899 nm. IR spectra were obtained using the ATR sampling technique in a Thermo Nicolet 6700 FT-IR spectrometer. The IR and UV-Vis data were visualized using Origin Pro 8.0 software. ^1^H NMR (400 MHz) experiments were performed with a Varian Inova spectrometer. Chemical shifts were referenced relative to TMS, and *J* values are given in Hz. The ^1^H NMR spectra were acquired by dissolving 6–8 mg of melanin in 0.8 mL NaOD in 40% D_2_O at 60 °C. The NMR data were processed and visualized using MestReNova software. HRESIMS data were recorded on a Thermo Q Exactive Plus mass spectrometer in positive detection mode. For this analysis, each melanin sample was dissolved in 300 μL of a mixture of 2 M KOH in MeOH/saturated NH_4_Cl aqueous/DMSO 1:1:1. Samples were directly infused in an Orbitrap instrument (Thermo Fisher Scientific).

### Detection of H_2_O_2_ in *U*. *maydis* by fluorescent microscopy

The parental and *UmRrm75* null mutant strains were grown in YEPD at 28 °C overnight. Cells were refreshed and grown until reaching an OD_600_ of 0.8–1.0. Subsequently, the strains were subjected to 15 °C, 28 °C or 37 °C for 4 h. For catalase treatment, cells were grown until reaching an OD_600_ of 0.8–1.0, after which 0 or 250 U/mL of CAT was added for 4 h. Then, 20 μM of 2,7-dichlorohydrofluorecein diacetate (DCFH2-DA) was added to each culture strain according to the protocol described by Fu *et al*.^61^. Cells were observed using an Axio Imager M2 microscope (Zeiss). Images shown are representative of the experiment conducted with three biological replicates. This experiment was repeated at least 3 times with similar results.

### H_2_O_2_ sensitivity assay, and H_2_O_2_ quantification in parental and *UmRrm75* null mutant strains

For the H_2_O_2_ sensitivity assay, the Δ*UmRrm75* mutant and parental strains were plated on CM medium. Filter disks were soaked with 1 µL of H_2_O_2_ (30% v/v) and placed on the center of plates. The halo sizes were measured from four biological replicates after 6 days of incubation at 28 °C. For each sample, four biological replicates (*n* = 4) were analyzed. Experiments were repeated at least twice with similar results. For H_2_O_2_ quantification, one gram (fresh weight) of FB2 parental and 1/46 null mutant cells (CFW) were collected. The cell mass was homogenized in 0.1% trichloroacetic acid and collected by centrifugation. Subsequently, cells were resuspended in 10 mM phosphate buffer (pH 7.0). Finally, 0.5 mL of 1 M potassium iodide (KI) was added. The samples were measured at a wavelength of 390 nm. Dilutions of a standard H_2_O_2_ solution were read for the calibration curve. Data of each sample were interpolated with the standard H_2_O_2_ curve and were reported as μmol/g of cell fresh weight (μmol/gCFW). For each sample, three biological replicates (*n* = 3) were analyzed. Experiments were repeated at least twice with similar results.

### Analysis of Δ*UmRrm75* mutants and parental strains by transmission electron microscopy (TEM)

The parental FB2, 1/2 and SG200, as well as null mutant strains 1/46, 1/40 and 1/53, were grown in MM liquid medium at 28 °C for 24 h. For the stress condition, strains were grown in 1 mM H_2_O_2_ and the cell pellet was collected. Cells were fixed in 3% glutaraldehyde for 2 h at room temperature, then washed 3 times with PBS and incubated for 4 h at 37 °C in a freshly prepared solution (5 mL) of 10 mg 3,3′-diaminobenzidine (DAB) in 0.1 M bicarbonate buffer (pH 10.5)^62^. Samples were postfixed with 2% OsO_4_ at RT for 1 h, washed with PBS, dehydrated with ethanol, embedded in Epon 812 Resin and polymerized for 24 h at 60 °C. Ultra-thin sections were obtained and contrasted with 4% uranyl acetate and Reynold´s lead citrate. Images were acquired with a JEOL JEM 1010 electron transmission microscope at accelerating voltages of 60 kV.

### Catalase enzymatic activity in FB2 and 1/46 mutant strains

The FB2 parental and 1/46 null mutant strains were grown in liquid MM at 28 °C for 10, 12 or 24 h. Protein extraction was performed as described by Hernández-Sánchez *et al*.^63^. Protein concentration was determined by the Bradford test^64^. Protein extract was used to quantify the enzymatic activity by the spectrophotometric method at a wavelength of 240 nm^65,66^. The CAT activity was normalized to the initial protein concentration and was expressed in U CAT/mg protein. For each sample, three biological replicates (*n* = 3) were analyzed. Experiments were repeated at least twice with similar results.

### Statistical analysis

Unpaired t test, One-Way ANOVA Analysis and Tukey’s post-test analyses were performed to assess statistical significance. GraphPad Prism version 5.0b (GraphPad Software, San Diego, California, USA) was used for the analysis. Data represent the mean ± SEM. Differences at *P* < 0.05 were considered to be significant.

## Supplementary information

Supplementary Information
