# Supplementary material for: The Ustilago maydis null mutant strains of the RNA-binding protein UmRrm75 accumulate hydrogen peroxide and melanin
Source: Sci Rep. 2019 Jul 25;9:10813. doi: 10.1038/s41598-019-47133-4 (PMC6658566; doi:10.1038/s41598-019-47133-4)
Supplement: Supplementary file 1 — Supplementary Information [file 41598_2019_47133_MOESM1_ESM.pdf]

The *Ustilago maydis* null mutant strains of the RNA-binding protein UmRrm75 accumulate hydrogen peroxide and melanin

Alma Laura Rodríguez-Piña[1], Margarita Juárez-Montiel[2], Itzell Eurídice Hernández-Sánchez[1], Aída Araceli Rodríguez-Hernández[1], Elihú Bautista[3], Alicia Becerra-Flora[1], Edgar Oliver López-Villegas[4] and Juan Francisco Jiménez-Bremont[1*]

[1]Laboratorio de Biología Molecular de Hongos y Plantas, División de Biología Molecular, Instituto Potosino de Investigación Científica y Tecnológica A.C., San Luis Potosí, México. [2]Laboratorio de Biología Molecular de Bacterias y Levaduras, Escuela Nacional de Ciencias Biológicas, Instituto Politécnico Nacional, México City, México. [3]CONACYT-CIIDZA, Instituto Potosino de Investigación Científica y Tecnológica A.C., San Luis Potosí, México. [4]Central de microscopía, Escuela Nacional de Ciencia Biológicas, Instituto Politécnico Nacional, México City, México.

[*] Corresponding author: Juan Francisco Jiménez Bremont email: jbremont@ipicyt.edu.mx

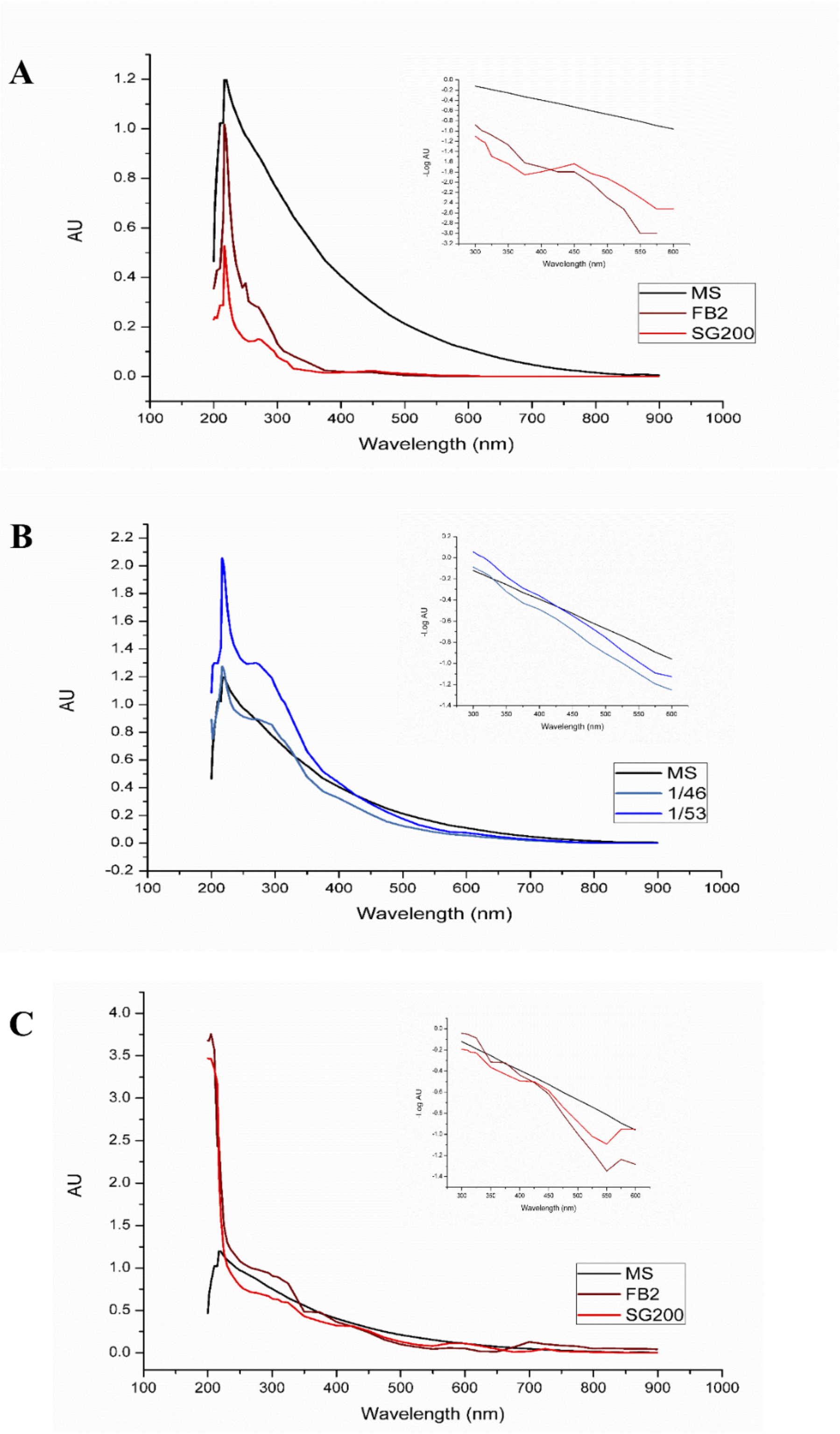

Supplementary Figure 1. UV spectra of melanin from parental and Δ*UmRrm75* null mutant strains

(**A-B**) UV-spectra of melanin pigments derived from FB2, SG200 parental and 1/46, 1/53 null mutant strains at. 28°C. (**C**) UV-spectra of melanin pigments from FB2 and SG200 parental strains at 37°C. The concentration of alkaline solution of melanin used was 10 µg/mL. Linear plots of optical density against wavelength represent purified melanin extracted from parental and Δ*UmRrm75* mutant strains in each condition.

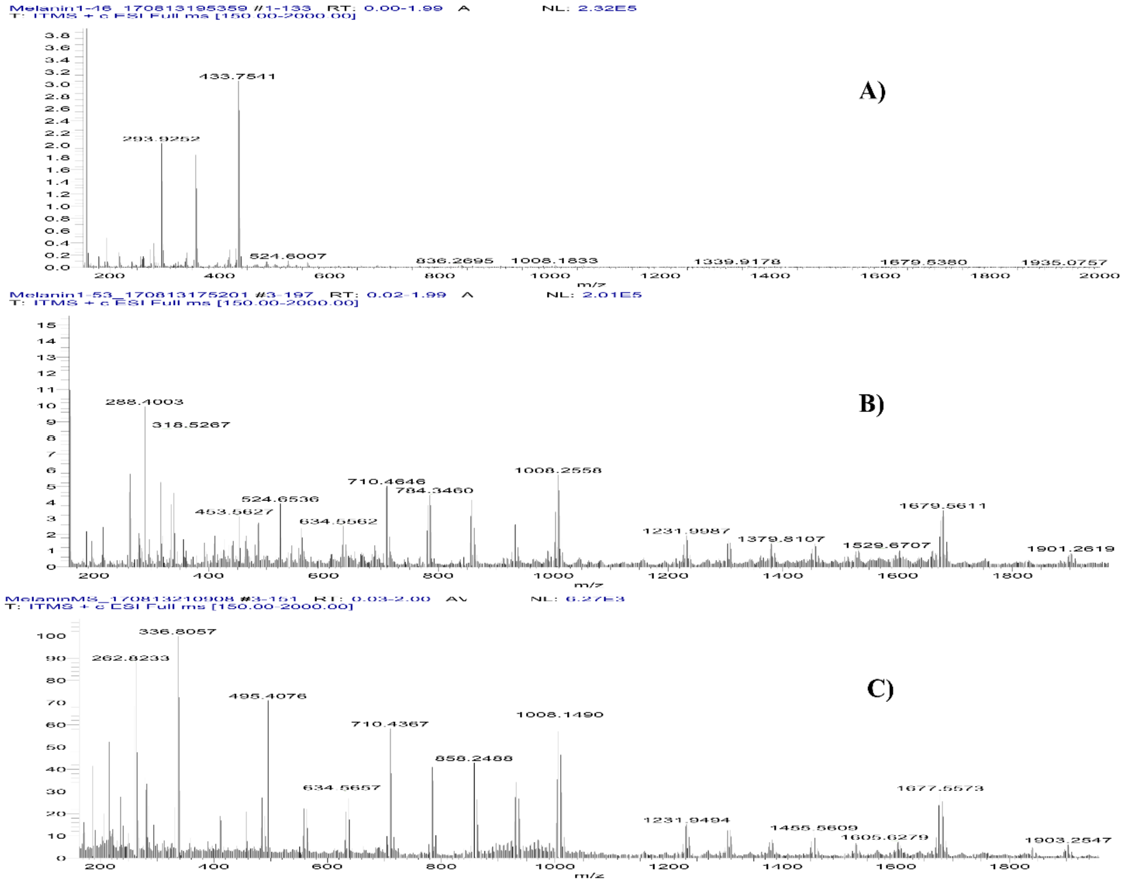

Supplementary Figure 2. ESI-MS analysis of pigments from Δ*UmRrm75* null mutant strains

ESI-MS spectra of melanin extracted from A) 1/46 null mutant strain (FB2 background), B) 1/53 null mutant strain (SG200 background) and C) spectra of synthetic melanin.

Supplementary Table 1. Strains of *U. maydis* used in this study

Strain	Relevant genotype	Source
1/2 (521)[a]	*a1b1*	Kronstad and Leong (1989)
2/9	*a2b2* (BX7A22 near isogenic to 1/2)	Gold et al. (1997)
FB2	*a2b2*	Banuett and Herskowitz (1989)
FB1	*a1b1*	Banuett and Herskowitz (1989)
SG200	*a1:mfa2 bE1bW2*	Müller et al. (1999)
MA-1/40	*a1b1* Δ*Umrrm75*	Rodríguez-Kessler et al. (2012)
MA-1/46	*a2b2* Δ*Umrrm75*	Rodríguez-Kessler et al. (2012)
MA-1/53	SG200 Δ*Umrrm75*	Rodríguez-Kessler et al. (2012)

[a] Strain 1/2 is strain 521, the sequenced strain of *U. maydis*.

Supplementary Table 2. Diagnostic melanin test in Δ*UmRrm75* null mutant and parental strains

**Temperature**	**28 °C**	**28 °C**	**37 °C**	
**Properties**	Melanin-Like-mutant strains	Melanin-Like-parental strains	Melanin-Like-parental strains	Synthetic melanin
Solubility in H_2O at 25°C	Insoluble	Insoluble	Insoluble	Insoluble
Solubility in organic solvents (chloroform, ethanol and acetone)	Insoluble	-	Insoluble	Insoluble
Solubility in Na_2CO_3	Soluble	-	Soluble	Soluble
Solubility in NaOH at 20°C	Insoluble	Insoluble	Insoluble	Insoluble
Solubility in KOH at 100°C	Soluble	Insoluble	Soluble	Soluble
Decolorization by oxidants agents (H_2O_2 30%)	Decolorized	-	Decolorized	Decolorized
Precipitation with HCL	Precipitated readily	-	Precipitated readily	Precipitated readily
Reaction for polyfenols with $FeCl_3$	Ppt.	Ppt.	Ppt.	Ppt.
Color Pigment	dark browm	yellow	dark browm	brown

Supplementary Table 3. Primers used in qRT-PCR analysis

Gene	F Primer	R Primer	Size (bp)
UmRrm75	5′-ATGCTCCCATGGTTGAAGTTTCGG-3′	5′-CTTGGGACAAATCTGAGCTGTACAC-3′	153
Yap1	5′-CATCTGCAGTTCCACTCCCT-3′	5′-GTGTGACAGTGTACCAGAGC-3′	165
Pex3	5′-ACCTGTCGTCCCATTCCAAC-3′	5′-TTCTGTGCGAATCTTCGACGTA-3′	160
GADPH	5′-CAACGACCCCTTCATTGACC-3′	5′-GGATGTTGGAGGGGTCCTT-3′	158
